# Implementing text-messaging to support and enhance delivery of health behavior change interventions in low- to middle-income countries: case study of the *Lifestyle Africa* intervention

**DOI:** 10.1186/s12889-023-16388-y

**Published:** 2023-08-10

**Authors:** Frank T. Materia, Joshua M. Smyth, Thandi Puoane, Lungiswa Tsolekile, Kathy Goggin, Stephen R. Kodish, Andrew T. Fox, Ken Resnicow, Scott Werntz, Delwyn Catley

**Affiliations:** 1 Division of Health Services and Outcomes Research, Children’s Mercy Kansas City, 2401 Gillham Road, Kansas City, MO 64108 USA; 2 Department of Biobehavioral Health, The Pennsylvania State University, University Park, PA USA; 3 Penn State College of Medicine, Milton S. Hershey Medical Center, Hershey, PA USA; 4 School of Public Health, University of the Western Cape, Cape Town, South Africa; 5 School of Medicine, University of Missouri – Kansas City, Kansas City, MO USA; 6 School of Pharmacy, University of Missouri – Kansas City, Kansas City, MO USA; 7 Department of Nutritional Sciences, The Pennsylvania State University, University Park, PA USA; 8 School of Public Health, University of Michigan, Ann Arbor, MI USA; 9 Agile Health Inc., Lincolnshire, IL USA; 10 Center for Children’s Healthy Lifestyles and Nutrition, Children’s Mercy Kansas City, Kansas City, MO USA

**Keywords:** mHealth, Text messaging, SMS, Health behavior change, LMIC, Diabetes prevention program, Community health worker, Implementation, Global health

## Abstract

The prevalence of non-communicable diseases, such as diabetes and cardiovascular disease, is rising in low- and middle-income countries (LMICs). Health behavior change (HBC) interventions such as the widely used Diabetes Prevention Program (DPP) are effective at reducing chronic disease risk, but have not been adapted for LMICs. Leveraging mobile health (mHealth) technology such as text messaging (SMS) to enhance reach and participant engagement with these interventions has great promise, yet we lack evidence-informed approaches to guide the integration of SMS specifically to support HBC interventions in LMIC contexts. To address this gap, we integrated guidance from the mHealth literature with expertise and first-hand experience to establish specific development steps for building and implementing SMS systems to support HBC programming in LMICs. Specifically, we provide real-world examples of each development step by describing our experience in designing and delivering an SMS system to support a culturally-adapted DPP designed for delivery in South Africa. We outline eight key SMS development steps, including: 1) determining if SMS is appropriate; 2) developing system architecture and programming; 3) developing theory-based messages; 4) developing SMS technology; 5) addressing international SMS delivery; 6) testing; 7) system training and technical support; and 8) cost considerations. We discuss lessons learned and extractable principles that may be of use to other mHealth and HBC researchers working in similar LMIC contexts.

**Trial registration **Clinicaltrials.gov, NCT03342274. Registered 10 November 2017.

## Background

The global burden of disease has shifted in recent decades from infectious diseases toward non-communicable, “lifestyle” diseases, particularly in low- and middle-income countries (LMICs). Health behavior change (HBC) interventions such as the Diabetes Prevention Program (DPP) have shown evidence in high income countries for reducing diabetes and cardiovascular disease risk [1–3]. These programs focus on encouraging healthy lifestyle changes, particularly weight loss through dietary changes and physical activity, usually through regular intervention sessions that are typically delivered in-person.

For these same programs to be similarly effective in LMICs they need to be adapted for resource-poor communities where many residents are low-income, have lower levels of education, have low health literacy, have limited access to transportation, and there exists limited capacity of health professionals to effectively deliver programs. LMICs also possess potential facilitators of HBC programs. For example, mobile phone use is widespread in LMICs [4, 5] and could be used within mobile health-based interventions (“mHealth” [6, 7]) to expand the reach of programs and increase the engagement of more vulnerable communities.

mHealth broadly entails the integration of portable and wireless technology (e.g., mobile phones) to enhance delivery of health interventions [8]. The multifaceted capabilities of mobile technologies can, for example, provide remote access to HBC program content, prompt participants to implement behavior change strategies, and provide on-demand reminders about program requirements. mHealth technologies allow interventions to extend interactions with program content beyond core intervention sessions, enhance participant motivation for meeting behavior change goals, and support program implementation. Studies in high-income countries generally support the utility of supplemental mHealth components to support HBC programs [9] but other considerations may be more salient in LMIC contexts.

There are limited evidence-informed guidelines in the literature for implementing mHealth in LMICs. A few “universal” toolkits and frameworks do exist, yet by design they are general in their direction and applicability across various mobile technologies and intervention applications. For example, the “mHealth Assessment and Planning for Scale Toolkit” (i.e., MAPS [10]) focuses on a set of suggestions (e.g., groundwork, partnerships, finances, operations, evaluation) to help guide mHealth development broadly. Also, Labrique et al. (2018) has more recently developed a set of “practical considerations for scaling digital health initiatives in LMICs” (e.g., program characteristics, human and technical factors, ecosystems [7]). A major limitation is that these extant guidelines neither provide specific guidance regarding the detailed nuances involved with selecting and developing text messaging for HBC nor do they present “short message service” (SMS; text messaging) as a single mHealth technology of choice. SMS is generally a wide-reaching, accessible, affordable, and popular mHealth methodology and may be best fit in various intervention-delivery scenarios for enhancing the reach and effectiveness of HBC programs in LMICs.

### Aim of this report

Our group recently completed a study in which we adapted the DPP for a low-resource community in South Africa (SA) utilizing community health workers (CHWs) together with a supplementary SMS system to support, extend, and enhance intervention delivery [11]. The study was a two-arm parallel group cluster RCT with crossover of the control arm. The work was conducted in Khayelitsha, a rapidly expanding urban district outside of Cape Town, South Africa. 99% of the residents are Black African, Xhosa-speaking, and face very high levels of poverty. Our team partnered with well-established NGOs in the area to deliver the intervention via trained CHW’s. CHW’s worked with existing health promoting community support groups (or “health clubs”) to conduct health screenings and delivering medications. Club members who were overweight/obese were invited to participate. The intervention was a seventeen-session in-person video-based curriculum directly adapted from the CDC’s National DPP program. A community advisory board helped guide our research team’s adaptation of the DPP, with all content, language, translations, and components designed to be culturally appropriate for the Xhosa-speaking residents of Khayelitsha. We have previously reported on the successful implementation of this program (“*Lifestyle Africa*”, Catley et al. 2020) [12] as well as a significant effect on HbA1C in a randomized trial (Catley et al. 2022) [13].

The primary aim of this report is to discuss the process that we used to design and implement an SMS system to support the seventeen in-person sessions of the *Lifestyle Africa* intervention, and to inform similar efforts. To do so, we draw upon our specific experiences in developing *Lifestyle Africa*, our broader expertise in mHealth design and implementation, and integrate these perspectives with broad guidelines in the field (e.g., WHO’s MAPS, Labrique’s “practical recommendations”). Accordingly, we identify eight development steps specific to SMS that we posit were crucial to successful development and implementation of the custom system in our South African study and that we believe forms a framework to develop other SMS-based mHealth intervention supports in other LMIC contexts. The development steps include: 1) determining if SMS is the appropriate mHealth approach; 2) developing the SMS system’s architecture and programming framework; 3) developing theory-based SMS message content; 4) developing SMS technology; 5) addressing specific requirements of international SMS delivery; 6) establishing testing procedures of the technology; 7) developing system training and technical support guidelines for staff managing the SMS system; and 8) ongoing monitoring and assessment of the cost of sustaining the SMS system for the duration of the study.

Below we describe the steps and illustrate our real-world experience implementing them to design the *Lifestyle Africa* SMS system. We also address some of the challenges of working internationally to deliver the program, as mHealth interventions lend themselves to delivery across the world and do not necessarily require systems to be housed in the country or region in which they are delivered.

## Main text

### Step 1: Determining if SMS is the appropriate mHealth approach

Researchers first must decide which type of mHealth approach (e.g., text messaging, mobile applications, mobile web, or eLearning) is most feasible for their intervention. One major factor affecting this decision is cellular connectivity in LMICs. “Second-generation” (i.e., 2G) cellular connectivity has existed since the early 1990s and supports basic functions such as voice calls and SMS. “Third generation” (i.e., 3G) cellular connectivity emerged in the 2000s and added internet connectivity to mobile devices, allowing for streaming of multimedia content and mobile applications (i.e., “apps”, [14]). 3G cellular infrastructure has expanded significantly in recent years, but is still inaccessible in some very rural areas [15]. Researchers must also consider if their target population mostly owns smartphones vs. basic “non-smart” cell phones [16], some of which do not fully support enhanced data capabilities [17].

Although SMS systems have fewer capabilities (e.g., limited multi-media, tracking, and content delivery options) than other mHealth approaches, SMS can nevertheless provide intervention support in key areas. For example, messaging can be designed to help participants retain curricular content and knowledge between intervention sessions. It can also provide motivation and enhance self-efficacy for HBC [6, 17]. The SMS system can also be designed to support and maintain contact with participants across extended periods of time without programming (e.g., holidays or logistical issues). Finally, text messages can be used to increase participant engagement and adherence by assisting with implementation planning and facilitating in-person participation (e.g., notifying participants which materials to bring to an in-person session, providing reminders about session dates).

#### Selection of SMS to supplement *Lifestyle Africa* intervention

An initial survey of potential study participants revealed that smartphone ownership was rare in our target population in South Africa, so 2G connectivity was judged to be the appropriate platform for reaching most of the participants. SMS, therefore, represented the best implementational option to achieve project goals. This was also consistent with evidence from the local context where studies have documented the potential of SMS for expanding health care access, facilitating health-promoting behaviors, and increasing participant adherence in community-based programs in SA [18, 19].

### Step 2: Developing the SMS system’s architecture and programming framework

System architecture and programming frameworks are helpful for SMS implementation planning as they can guide researchers in determining different parameters for the way messages should be sent to participants. A message architecture specifies aspects such as the message types (e.g., texts sent to prepare participants for the program, texts sent to remind participants of the upcoming session, texts sent after intervention sessions to reinforce content), timing and scheduling of message delivery, the volume of messages, personalization and any other advanced programming or logic considerations. These frameworks should be broadly robust (i.e., applicable across different users, sites, and adaptable to idiosyncratic disruptions such as different schedules for different groups) and not produce additional barriers to access (e.g., not sending messages at inconvenient times, not sending an overwhelming number of messages),

The first SMS system decision researchers should make is whether it should function as a two-way or one-way messaging program. In two-way systems, participants not only receive program messaging to their mobile devices, but they are supported to text back questions and receive individual attention and responses from program staff assigned to monitor the system (e.g., CHWs) or advanced chatbots (automated systems that generate replies, often based on keyword content). One-way SMS systems only support communications to be sent to participants; participants may text into the program, but in a one-way system, inbound communications are not monitored in real time (or at all) and participants would generally receive no replies to their text messages. Deciding upon implementing a two-way vs. one-way system involves discussion around both pragmatism (e.g., does the project have the available resources and staff to support consistent two-way monitoring and replies?) and need (e.g., does the population and behavior require ongoing interactive support to achieve targeted outcomes?).

Attrition and retention are key indicators of the system’s implementational success, and conceptualization and measurement of these outcomes may differ depending on system architecture – specifically, whether a one-way or two-way system is built. For both types of systems, if a “STOP” command is built in (i.e., a participant can reply “STOP” at any time to leave the SMS program), tracking the number of participants who opt out is one metric for attrition. Regarding retention, most SMS systems will allow researchers to identify if participants are successfully receiving SMS messages to their phones. For one-way systems, retention can be measured over time by tracking how many messages are successfully delivered between and within individual participants. For two-way systems that generally elicit dialogue between a coach and participant or require the participant to reply to particular messages, participant responses can be tracked as a measure of retention. Overall, to mitigate attrition and support retention with the SMS system, a research team member (e.g., a CHW) should be tasked with reaching out to participants who are either not receiving their SMS messages or not responding as requested. In some cases technical assistance may be necessary (see “Developing training and technical assistance frameworks” section below).

As a general rule of thumb when considering system architecture, researchers must ensure potential participants will find the SMS system’s programming logic (i.e., timing, volume of SMS) to be culturally appropriate to their needs and preferences. One way to ensure system architecture matches the target population’s preferences is to seek the help of a community advisory board (CAB) made up of multiple stakeholders from the target community (e.g., local residents and community leaders) who can assist researchers by making system programming recommendations regarding timing and frequency that would be acceptable to target participants [20]. Feedback from the research team and from the CAB should also be sought out to make decisions on the different types of messages that will be sent.

Researchers should also consider specific requirements of the intervention program that the SMS system is designed to support in terms of “types” of messages and timing. For example, is it sufficient to send messages from a single bank of messages (i.e., a single type) on a consistent schedule over time (e.g., 2 weekly messages from the bank for 8 weeks)? Alternatively, some intervention programs may require different types of messages to be sent at different times and at different frequencies (e.g., there may be messages that need to be sent in the early phase of the program that are different from messages that need to be sent at a later phase and/or messages may need to be sent more frequently initially and then taper). Based on these kinds of considerations, the types of messages and the schedule may be quite simple or very complex to design. Researchers should also consider the possibility of potential schedule disruptions and how any time-based message system will adapt to those scenarios.

In many LMIC contexts, program participants may be close friends, neighbors, or family members living within the same household or community—and participants may share their individual text messages with others. This prompts another level of consideration of the system’s architecture – the degree of personalization integrated into SMS logic and content (e.g., including participants’ names, tailoring content to individuals’ data). With close cross-participant interaction in mind, messages may be more impactful if some elements of personalization are adopted, which may enhance participants’ internalized feelings of program “fit”, commitment, and engagement [21]. For SMS programs that intend for all participants to receive the same “dosage” and library of messages, however, it may be necessary to avoid personalization strategies that would result in differential treatment (e.g., participants receiving different numbers of messages, or messages with different content).

Researchers may choose to adopt an SMS framework that incorporates an individualized “tailoring” process. This strategy from behavioral science is intended to enhance motivation and engagement with intervention content by making participants feel individually supported in achieving program goals and having a program that is “personalized” for them [21]. Text messages sent to individual participants can include tailored data that is housed in the SMS system’s backend, such as participant name, their specific behavioral goals and program progress, and other ongoing tracking or monitoring information such as step counts or weight [22–24]. It is not always feasible, however, to integrate personalized information like this into message programming (e.g., due to financial constraints for building this level of technology, staffing constraints in finding time to update such data). In this case, researchers can still develop a framework that mimics a tailoring process for close-knit participants. For example, a simple randomization programming scheme can make the order of all SMS message receipts different for each participant. In this approach, participants still receive equivalent treatment (in content and ‘dose’ of SMS), as all participants receive the same messages (and hence the same level of exposure to intervention content); but in this programming scheme, messages can be delivered in random orders and at random times within segmented parts of the days that make the participant SMS experience feel less homogenous, specifically in the event they share their messages with another participant.

#### Lifestyle Africa SMS system architecture and programming framework

After careful consideration of resources available to support the SMS system, the research team decided that it was more pragmatic to design the system architecture and programming framework as being a one-way SMS delivery system. *Lifestyle Africa* program staff were already significantly burdened with program responsibilities and there were no resources to hire additional staff to monitor and respond to participant text messages so a two-way SMS system was deemed not feasible and likely an impediment to program scalability. Had it been possible to engage additional staff on SMS system implementation and management, we would have considered designing the system to be two-way. In this scenario, staff members would have served as “SMS coaches”, answering program questions in real-time via SMS and providing tailored behavioral supports to participants as they went about their daily routines practicing the skills they learned in the last in-person *Lifestyle Africa* session.

The same CAB that was providing guidance on the seventeen-session *Lifestyle Africa* curriculum was engaged in the SMS planning and development process. This advisory board was made up of multiple stakeholders including local residents living with diabetes, local residents with expertise in diabetes, and other community leaders (e.g., representative of the provincial SA Department of Health, a community elder). The CAB indicated that most participants had employment, family, or healthcare obligations between late morning and late afternoon. The CAB also noted that three or more messages a day would likely feel like an “overreach” and become an annoyance to community members. The team used this feedback to create a two-message per day programming scheme, with the first text being sent early morning (i.e., between 9 am and 11am) before participants generally left their homes for the day and again in the early evening (i.e., between 5 pm and 7 pm) after participants had likely finished supper and were home for the night.

**Fig. 1:**
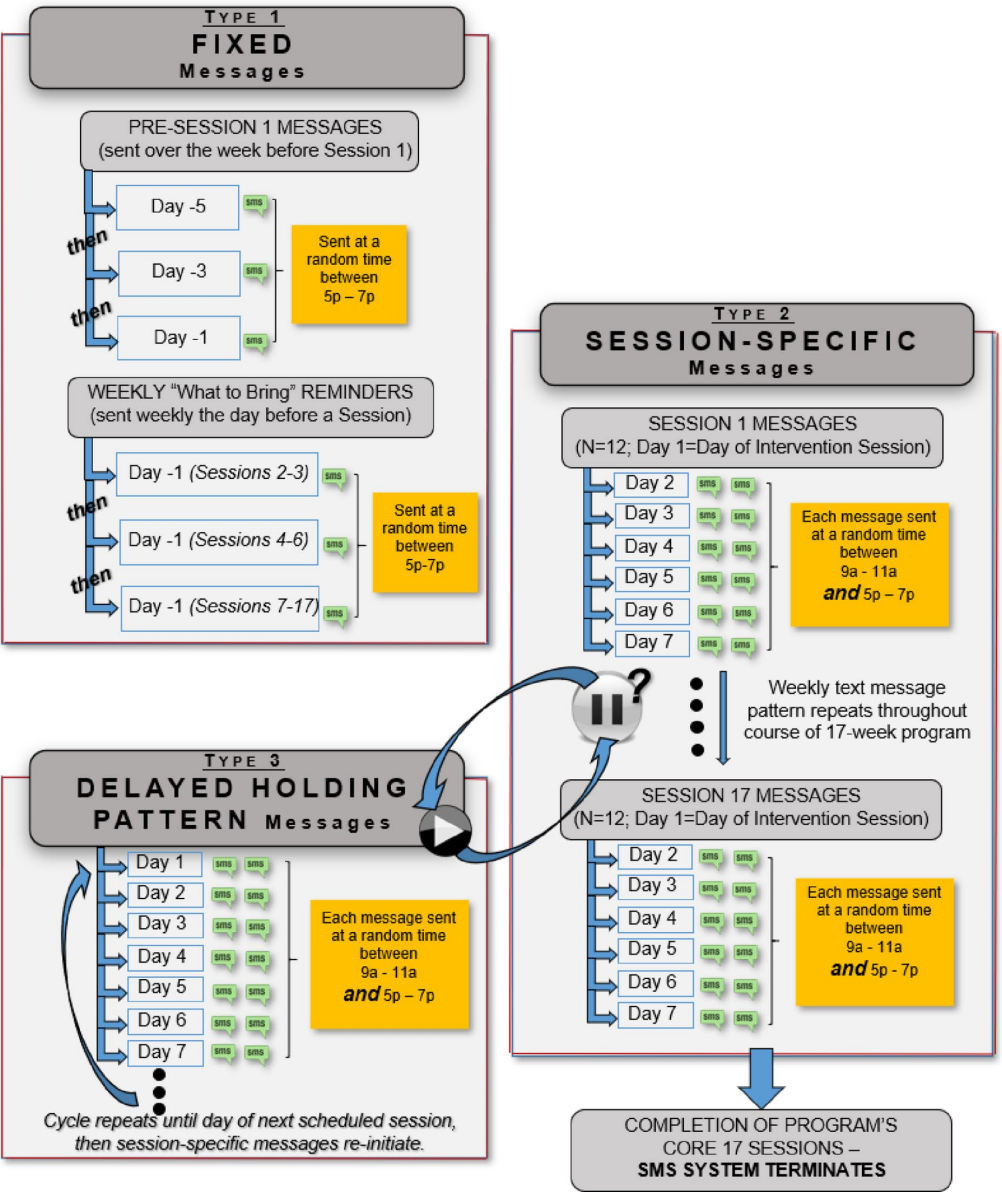
Lifestyle Africa SMS system architecture and programming framework

See Fig. 1. The final SMS architecture and programming framework outlined three SMS types, timing, and scheduling of messages:

- Type 1 consisted of “**fixed messages**”. Fixed messages were sent five days, three days, and one day before session 1 to excite participants and encourage them to come to the first *Lifestyle* Africa in-person session. Fixed messages were also sent the evening before each of the following seventeen weekly intervention sessions reminding participants about coming to the in-person program and which specific materials to bring. The intent was that fixed messages would not only facilitate attendance and preparation, but also support staff by reducing the need to personally communicate with each participant individually each week. Although personal calls from a staff member may be more effective than a text message for encouraging program attendance, the incremental gain of a phone call over a simple pre-programmed SMS was not sufficient to warrant the labor required.
- Type 2 consisted of “**session-specific messages**” sent twice a day at a random time to each participant between 9 am and 11am, and then again at a random time between 5 pm and 7 pm. Session-specific messages were sent on the six days between weekly scheduled intervention sessions. These messages were intended to enhance and extend programming by continuing to reinforce the concepts learned the last time participants met in-person, and supporting participants in enacting skills in their daily lives that were assigned as “homework” or skill practice. For example, after a participant attended Session 2 on a Tuesday, they received session-specific messages for Session 2’s curriculum from Wednesday through the next Tuesday’s session (Session 3); thereafter, the SMS programming logic delivered session-specific messages between sessions for the entire program. All participants received the same session-specific messages throughout the week, except each participant received their SMS in a random order to emulate an individualized tailoring process (in the event they shared their messages with close others in the program).
- Type 3 consisted of generic “**delayed holding pattern**” messages that could be sent in the event a class was cancelled until programming resumed. For example, in our SA community, businesses shut down for multiple weeks in December and January in observation of holidays. In addition, during the trial there were also multiple bouts of inclement weather that required rescheduling of weekly sessions. We addressed this by including a “pause” function in our SMS system that postponed sending of session-specific messages until the missed in-person session was able to be held. Continuing contact with program participants during “paused” or “off” time from the program was important in order to maintain interest and engagement over long periods without in-person meetings. To this end, a set of generic “delayed holding pattern” messages (i.e., not tied to session content) were sent as general support (e.g., broad motivational messages encouraging healthy eating, general reminders to track exercise) during weeks where programming was postponed or paused and no new session-specific content was being introduced. Upon resuming normal programming, these delayed holding pattern messages ceased, and the system returned to its normal schedule and scheme for sending session-specific message flows.

### Step 3: Developing theory-based SMS content for HBC interventions

A crucial step in developing the SMS system is developing a library of SMS messages with a sufficient number of messages to match program needs. A helpful starting point may be reviewing the program’s core components and contents (e.g., printed resources such as workbooks or facilitator guides) to identify targeted content areas across the various functional message types. Principles of thematic analysis [25] can be used to examine the intervention in its entirety and identify general content areas. Generally, it is key for the research team to identify and prioritize focusing content areas around modifiable behaviors that can necessitate change to improve the health outcomes targeted by the intervention program before crafting messages. Some common content areas might include physical activity, healthy eating, tracking (i.e., self-monitoring), stress management, social support, or behavioral maintenance.

Messages should also be developed based on empirically supported principles of health behavior change. There are over fifty theoretical frameworks in health communication and behavioral science to choose from [26] (e.g., the Theory of Planned Behavior [27], The Health Belief Model [28], and the Persuasive Health Message Framework [29]) that can be used to identify key constructs to guide message development. For example, the bank of messages for a particular message type might encompass messages from a variety of theoretical constructs or categories such as implementation planning, motivation, or self-efficacy. The main recommendation is that research teams develop or use messages with some theoretical grounding to ensure that collectively the messages support behavior change in a coherent and empirically grounded manner.

Working backwards from the established content areas and “categories” of messages, the research team can develop individual messages that fit within the predetermined types and categories. The number of messages needed within each content area and category will have also been predetermined by the agreed upon system architecture and programming framework (i.e., determined by such factors as the number of messages to be sent per day and the duration of the program in days or sessions). The entire message library should be reviewed by the research team to ensure the messages accurately match core program content and reinforce strategies from behavioral science for supporting HBC. In addition, steps (e.g., formative research to inform message development; pilot testing of messages) may be taken to ensure that messages are reviewed to ensure they adhere to social and cultural norms of participants.

Depending on the situation it may also be necessary to develop messages in one language (e.g., that of the research team) that then need to be translated to another language (e.g., that of the local community). We strongly recommend that any such message translation should be conducted by bilingual, bicultural research team members or hired professionals, and translation should be cross-checked using an inter-rater method, such that all messages are iteratively reviewed twice by two different native-speaking translators.

#### Lifestyle Africa SMS content development

The research team completed multiple iterations of message drafting, development, and translation with the SA *Lifestyle Africa* CAB, as well as with bi-lingual SA research staff. A complete *Lifestyle Africa* message library of 336 messages across the three different predetermined types was developed in English, and these messages were carefully stratified by *Lifestyle Africa* curricular content recommendation (physical activity, healthy eating, tracking, shopping/cooking, stress management, mental health, social support, reducing barriers to change, and behavioral maintenance). The *Lifestyle Africa* research team members had extensive training and previous intervention development successes working with the Theory of Planned Behavior (TPB [28]) and the Health Belief Model (HBM [27]) to develop HBC content. These two theories focus broadly on addressing individuals’ perceptions, cues, and self-efficacy for HBC, and behavioral intentions and have been extensively applied to weight related behavior change [30, 31]. Therefore, messages were evenly distributed across five categories that were selected and based broadly on constructs from the TPB and HBM (reminders, implementation planning, motivation, self-efficacy, and affirmation). Messages were organized into an easy-to-use spreadsheet with different tabs for each week of the *Lifestyle Africa* intervention. Messages were then translated by bilingual bicultural team members using a two-step process in which one person translated the message and a second person independently reviewed the translated message. Members of our CAB were then asked to review the entire SMS library to ensure the language was aligned with the local vernacular and matched the cultural and social norms of participants. They were also encouraged to make suggestions for improvement (e.g., if words and sentences seemed too long or sounded too formal or informal). Table 1 displays exemplar messages.

**Table 1:** Lifestyle Africa SMS message library – English content examples with Xhosa translations

**FIXED MESSAGE EXAMPLES**		
**Message Type**	**English Content**	**Xhosa Translation**
Pre-session 1 Introductory Message	Well-done taking the first step to living a healthier, more active lifestyle by joining Sivile Senza!	Wenze kakuhle ngokuthatha inyathelo lokuqala lokuphila ngokusempilweni, nokuzilolonga kakhulu ngokungena kuSivile Senza
“What do bring” Materials Reminder	Remember to bring your notebook, activity log, and food log this week to Sivile Senza	Ungalibali ukuphatha incwadi yakho kuleveki xa usiza kuSivile Senza
**SESSION-SPECIFIC MESSAGE Examples (taken from Session 3, "track your activity”)**		
**Message Category**	**English Content**	**Xhosa Translation**
Reminders	Household chores like sweeping and mopping – when done at a moderate pace – are good activities	imisebenzi yasendlini enje ngokutshayela nokucoca phantsi-xa uzenza ngesantya esiphakathi-yindlela yokuzilolonga elungileyo
Implementation Planning	Using a watch or watching the clock will help you count your minutes when tracking!	Ukusebenzisa iwatch okanye ukujonga kwiwatch yedonga kungaluncedo ukwazi ukubala imizuzu yakho xa ulandelela!
Motivational	Want to see the number on the scale go down next week? Track your progress toward your activity goal!	Ufuna ukubona inani lisihla esikalini kuleveki izayo? Landelela inkqubela yakho yeenjongo zakho zokuzilolonga!
Self-Efficacy	Now that you are tracking your activity, you may be able to think of parts of your day when you can add activity!	Kuba ngoku ulandelela imithambo yakho, ungakwazi ukucinga ngamanye amaxesha osuku lwakho onokongeza ngawo ukuzilolonga
Affirmation	When you track your activity and see that you are less active, you have the power to change that pattern!	Xa ulandelela imithambo yakho uze ubone ukuba uwuzilolongi ngokwaneleyo, unako ukuyitshintsha loo nto!
**DELAYED HOLDING PATTERN MESSAGE Examples:**		
**Message Category**	**English Content**	**Xhosa Translation**
Implementation Planning	You don’t have to make big changes. Take small steps to achieving your health goal	Uwunyanzelekanga ukuba wenze utshintsho olukhulu. Thatha amanyathelo amancinci ukufikelela kwinjongo yakho yempilo
Motivational	Remember, you will feel good and your body will be healthier after eating a healthy meal	Khumbula, uzakuziva kamnandi kwaye umzimba wakho uzakubasempilweni emva kokutya isidlo esisempilweni

### Step 4: Developing SMS technology

Assuming that researchers are not developing their technology themselves, they will need to identify technology firms with whom to work; ideally, it is desirable to work with providers who are sensitive to and experienced with the nuances of working with academic researchers in international contexts. Finding such providers is typically accomplished through word-of-mouth recommendations from other scientific investigators or practitioners who previously implemented SMS systems in LMICs, as well as other efforts (e.g., literature review, internet searches). Discussions and negotiations with multiple companies can lead to identification of the right technology developer. Conducting a series of early meetings with the selected developer, during which the research team shares their SMS system architecture and programming framework and drafts of messages (even if not in final form) will help to determine the scope of the project and pricing. This is particularly important for determining the project timeline and ensuring the SMS system is programmed, tested, and fully functioning by the time the intervention study is scheduled to begin. Researchers can negotiate pricing for SMS development and implementation services and should finally agree upon a price that fits their budget.

The technology developer then builds the web-based SMS management system. Ongoing (e.g., weekly) meetings with the research team help to ensure open communication with the developers as they prototype multiple versions of the SMS management system, which are typically accessed via a website portal. A web-based system is likely preferred (as opposed to a system using native computer applications) so that members of the research team in different geographic locations can manage and monitor SMS activity from any device with a web browser and internet.

The developer will also assist the research team in deciding if their SMS communications should be sent from a “short” 5-digit code, or a “long” 10-digit code that appears more like a traditional phone number. For participants to be able to identify SMS communications from the program, a dedicated code or number must be secured. This allows program participants to always receive messages from the program from the same number, and allows them to easily find previous messages on their mobile phones.

#### Lifestyle Africa SMS technology development

The research team met with and vetted multiple US and SA technology companies to describe the project’s SMS development and programming needs and obtain quotes. The research team eventually decided to contract with a US-based company that had prior experience and vested interest in academic research and HBC via SMS. Specifically, the company had advertised on their website a previous partnership with US academic researchers to implement an HBC text messaging program in a foreign country. During initial meetings with the company, the CEO provided an in-depth overview of how their team approached international SMS campaigns with academic partners in the past and provided some initial “ballpark” thoughts on pricing for the project. Given this company’s well-aligned experience and their proposal of a price within the project’s budget, the research team quickly decided to hire and execute a contract.

Early in the timeline of the overall project, multiple months of weekly planning meetings between the company’s project managers, engineers, and *Lifestyle Africa* research staff provided the company with all the relevant information needed to design the SMS system. A draft of the SMS library was sent to the company, as well as an outline of the system architecture and programming framework. Between these meetings and these materials, the technology company was able to successfully develop the system to meet the project’s needs in approximately five months.

The developer designed a web-based system that allowed for enrollment of program participants and their personal mobile phone numbers into the SMS program. Because there were multiple program locations with associated cohorts moving through the program at somewhat different points in time, the systems allowed for tracking of each location’s progress throughout the program; this ensured the appropriate session-specific and reminder messages were delivered to the relevant participants in sync with their location’s progress through the curriculum.

The technology company also walked the research team through the pros and cons of using a 5-digit “short code” versus a 10-digit phone number. For example, some participants’ mobile devices may block 5-digit short code messages, misidentifying them for spam messages. On the other hand, participants may mistake messages from 10-digit numbers as coming from a person with the wrong phone number instead of the program. The CAB was consulted and indicated that most local participants would be more familiar with short codes, so researchers decided to select a 5-digit short code as the sender number from which *Lifestyle Africa* participants received SMS. The technology company helped the research team develop language for participants around recognizing the program’s short code and unblocking any receipt issues.

### Step 5: International SMS implementation

If the technology developer is based outside of the country in which the program is being delivered, it is likely the servers and technology that host the web-based SMS system are also located in the country in which the platform is developed. Establishing a “gateway” avoids considerable costs to the program and possibly to the recipient of sending international text messages. A gateway company with international access to the LMIC’s cellular networks is therefore most times necessary to facilitate sending messages from the originating country to mobile phones overseas to help control costs and ensure direct contact with international participants. The SMS system’s domestic platform and the gateway company must have compatible “application programming interfaces” (i.e., APIs) to establish a successful connection. It is worth noting that some particular countries require the SMS platform to be housed within the country (or region) to which the messages are being delivered to protect participants’ data and ensure privacy (e.g. E.U. requirements as part of general data protection legislation). To work around such requirements, system integrations can still be accomplished by deploying the solution in cloud computing systems that can be housed and maintained in authorized regions based on local privacy legislation.

The SMS technology developer spearheads identification, vetting, and recommendations to the research team on cost-effective and reliable international gateway providers in the LMIC of interest (note that we discuss more details about the costs in the “Costs” section below). Once a gateway collaboration is established and company hired, the technology developer then works with the gateway to establish the SMS system’s code for participant communications [32]. The gateway provider will provide a list of codes currently available in the country of interest that the research team can choose from and then dedicate the code to the system.

#### Lifestyle Africa international SMS implementation

**Fig. 2:**
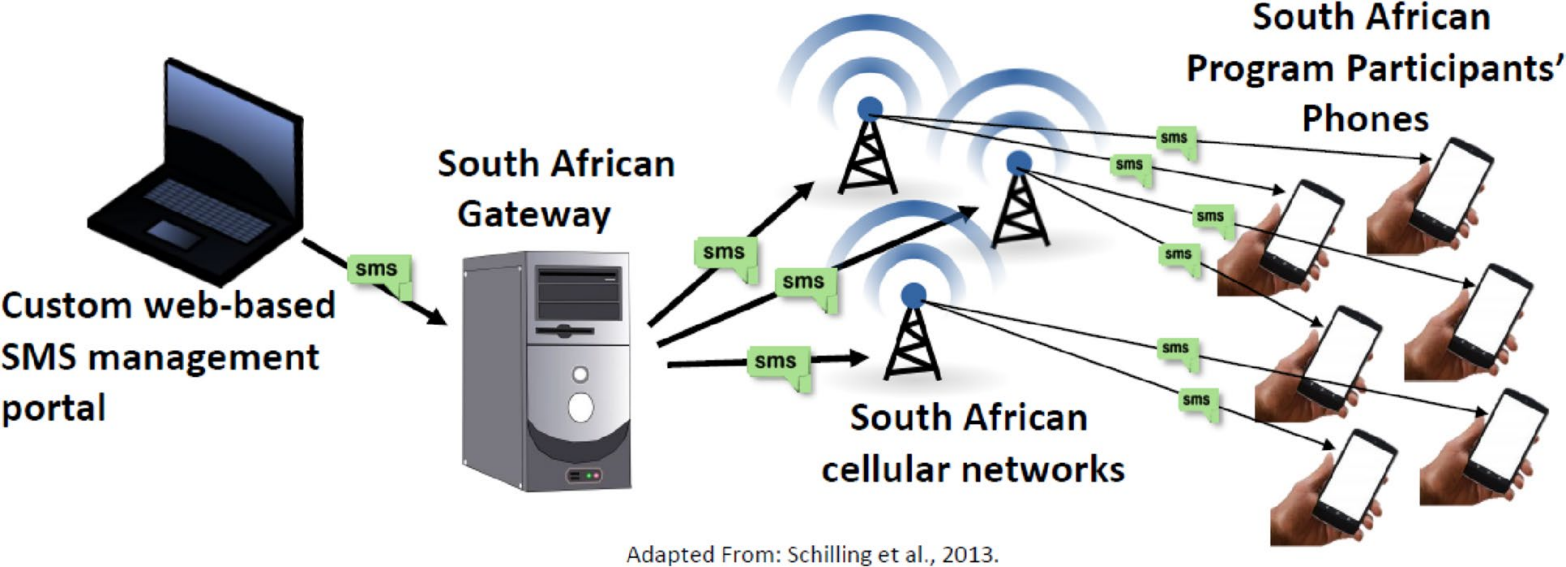
Lifestyle Africa domestic system to international cellular gateway

The US-based technology developer explored multiple international SMS gateway companies in SA, their individual pricing structures, and other factors such as security standards. A recommendation was made to partner with a company that was affordable, had a positive reputation for customer support, and most importantly used a technology platform that could successfully connect with the SMS system’s API to successfully transfer messages from the US platform to the SA gateway (and ultimately out to SA cellular providers and *Lifestyle Africa* participants’ mobile phones). See Fig. 2 for a graphical depiction of international gateway integration.

### Step 6: SMS system testing

After the web-based SMS management system is developed, testing procedures are necessary to ensure the system is working as intended before real-world implementation with program participants [32–34]. The first step involves identification of individuals involved with the project who can serve as mock participants to test the system. The system should be tested by individuals present in all involved countries including the LMIC to ensure the international gateway is working appropriately and that SMS messages are successfully being delivered internationally at the appropriate timing, frequency, and randomization schemes.

Individuals who help test the system should be enrolled in the SMS system and surveyed periodically during the testing process to report on the days, times, and types of messages they are receiving. Ideally, system messaging should be tested with a phone from each of the major carriers within the destination country. System fidelity can be evaluated by cross-checking tester responses to back-end data in the web-based SMS system to ensure matching between system function and the SMS architecture and programming framework. In some situations during testing, issues with system performance may be detected, such as international testers receiving messages at the incorrect times of the day or not at all. The research team must keep detailed records of these errors, collate them frequently (daily or a few times a week may be most prudent), and report them back to the technology developer, who can address the problems. The technology developer can provide specific instructions for diagnosing issues during testing, any standard troubleshooting suggestions, and their preferred method and frequency for being notified of system bugs.

#### Lifestyle Africa SMS system testing

With the help of the domestic technology developer, system testing was conducted at different rates and with different testers, allowing some testers to go through the entire seventeen-week *Lifestyle Africa* SMS curriculum’s flow, while other testers were only enrolled for a select number of sessions. Multiple weeks of iterative testing revealed a few minor system errors. Research staff who identified the issues first tried to pinpoint the problem via internal troubleshooting. If the research staff was unable to alleviate the problem, the issue was triaged to the technology developer. If there was a programming issue on the SA gateway’s end of the system, the technology developer worked to alleviate the issue, then reported back to the researchers that the problem was resolved. This provided for rapid response and resolution to most issues and had a clear communication process.

One example of an issue that arose during testing was that one tester was not receiving any messages. After working with the technology developers, the research team determined that the tester’s phone number was entered incorrectly during SMS enrollment. Another example of an issue was that during one particular week, multiple testers reported not receiving their text messages at the correct time. The research team worked with the technology developer and identified an issue in discrepancy between US daylight savings time and overseas time zoning. The issue was quickly and easily addressed by the technology developer.

### Step 7: Developing training and technical assistance frameworks

Given the possibility of errors of various sorts (e.g., user, technological) throughout the intervention delivery period, we suggest that researchers identify a team of “SMS managers” – individuals tasked with day-to-day management of the web-based SMS system concurrent with core intervention programming. SMS managers’ general responsibilities might include, by way of example, enrolling new participants into the SMS system and monitoring SMS delivery schedules to match core programming. SMS managers can also periodically check that the messages participants at each location are programmed to receive match the planned SMS protocol with respect to sequence, frequency, and timing.

The research team should consider developing training materials in different formats to familiarize SMS managers with the web-based interface and ensure they understand how to implement the system as intended. These might include video tutorials that show step-by-step on-screen recordings of using the SMS web-based management system, and manualized guides with screenshots and FAQs. The researchers also should consider hosting multiple live (in-person or video-based conference calls) with SMS managers to answer questions and conduct real-time demonstrations to ensure they are able to demonstrate functional competence with the SMS interface and system management responsibilities. The technology provider can assist in developing training materials and facilitating training sessions.

Unexpected problems normally emerge despite extensive testing. For example, it is not always possible to account for how extreme weather, internet connectivity, or power supply issues in either the host country or LMIC location may create system sending or receipt issues. As another example, wireless network carriers may change their guidelines without notice regarding how they process short-code and 10-digit code SMS, which might interrupt message delivery to participants. As such, we believe it very helpful to develop a chain of real-time technical support for SMS managers. Given possible time zone differences between the research team and the LMIC location, having some mechanism of support for SMS managers (ideally a direct emergency contact at the technology developer) that they can access any time is important for prompt reporting and response to issues with SMS.

#### Lifestyle Africa SMS system training and technical assistance

The research team developed a variety of materials to assist in training SA and domestic SMS managers for enrolling participants and monitoring the system. This included extensive training manuals that the domestic technology developer helped create, printed FAQ sheets, and step-by-step troubleshooting guides. Web-based training videos were developed and hosted on a private cloud-based service as a primary training tool for SMS managers to watch. Web-based video was selected, as in-person training of SMS managers in SA was not possible from afar in the US; even live-streaming video calls needed to be minimized because they were hampered by significantly different time zone differences and internet stability issues.

A chain of technical assistance was implemented so SMS managers would have real-time help if any problems emerged with the system. SMS managers would reach out to a dedicated email or phone number monitored by research team staff. If research staff could not alleviate the problem, they would triage the issue to the technology developer, who provided proactive technical assistance to address questions very quickly (most times within a few hours). In the event a particular program group had to cancel or postpone a session, SMS managers were also in charge of activating the “delayed holding pattern” generic messages for that specific group, and then re-starting session specific messages when normal programming resumed.

### Step 8: Cost monitoring and assessment

Researchers must ensure that the budget allocated to support SMS implementation is enough to sustain the cost of maintaining the system throughout the duration of the intervention program. The SMS technology developer should help to guide budget planning at the beginning of the project and inform researchers of all costs that could be anticipated throughout the project. On top of initial development costs (i.e., paying the technology developer to build and assist with testing of the technology), other financial obligations may include: paying a nominal flat fee for each SMS message sent (which can be purchased in batches as needed throughout the study from the gateway company); possible rental fees (commonly charged annually, bi-annually, or quarterly) by the gateway company to retain possession of the dedicated phone number used for participant SMS communication; and possible bi-annual renewal fees to the gateway company to maintain constant access to the LMIC’s cellular network providers. Most SMS technology developers should support monitoring of fees, purchasing SMS messages and rental/renewal costs as needed throughout the project, and then invoicing all necessary purchases back to the research team.

Continued monitoring and assessment of cost should be an ongoing discussion point for the research team. For example, if researchers want to add a new feature late in the development process, there may be cost overruns. If during the program there are high attrition rates, more SMS messages will need to be purchased from the gateway than originally budgeted for. Staffing and participant support costs may end up requiring more resources than originally anticipated. The recommendation here, generally, is to consider cost thoughtfully within the research team and with the developer, and to think and plan broadly for possible scenarios that may result in unplanned charges or extra fees.

#### Lifestyle Africa SMS costs

The total cost of building the SMS system, as quoted by the technology developer, was within the research team’s budget. The research team’s institution was billed monthly for SMS implementation and support fees. The SMS developer paid up front costs to rent the project’s dedicated short code from the SA gateway, as well as the gateways’ annual renewal fee and the minimal cost per SMS message sent. The technology developer invoiced these costs back to the research team’s institution for reimbursement. These cost data were carefully recorded by the research team in order to conduct a cost–benefit analysis of the overall program. More specific analyses of the implementation costs associated with the *Lifestyle Africa* trial are reported elsewhere (see Catley et al. [13]).

## Conclusions

Given the rising prevalence of non-communicable disease and lifestyle-related illnesses in LMICs, there is burgeoning need for research on how mHealth implementation can support and enhance the effectiveness of health programs through greater reach and tailored content to vulnerable communities. The primary purpose of this report was to disseminate to other researchers working in similar contexts our proposed development steps for building custom SMS systems, as well as the specific factors and decisions that the *Lifestyle Africa* research team worked through. The development and implementation recommendations provided in this report align with a number of the broad mHealth tenets from WHO and Labrique and colleagues (e.g., iterative design approaches, technology considerations, formative research with a community advisory board) but provide information specific to text messaging and also describe a real-world example of our application of this guidance. For example, the existing broad mHealth in LMIC recommendations suggest direct user-centered research with participants to define architecture and content preferences. In some instances to save resources, researchers may instead use relevant partners (i.e., a CAB and research staff with significant familiarity with target population norms) as proxies to inform culturally appropriate approaches to SMS design and implementation. As another example, WHO and Labrique discuss training of end-users; however, researchers may not always find it necessary to direct participants on how to send and receive text messages [7, 10]. Overall, the considerations of mHealth technology development for an international SMS system (e.g., gateways, testing) are unique and differ for developing an SMS system rather than, for example, a mobile app.

These SMS development steps were presented in what we hoped was a logical order; in real-world practice, however, these steps are not necessarily addressed in this specific order. An iterative design and testing process can be utilized [10, 32, 33]. As one area of system development nears completion, it can be evaluated for efficacy (for its intended purpose) and re-developed if not working as needed, all while other areas of SMS work are in different development and redevelopment stages.

Our suggested recommendations provide additional ideas with unique detail specific to SMS. We broadly recommend researchers be focused on creating mobile SMS messaging that is highly aligned with tenets of the program’s core curriculum so that messages bolster exposure to the therapeutic components of the intervention. Messaging should also be developed using theoretically sound methods from behavioral science to ensure messaging strategies are evidence-based, and therefore more likely to effectively support HBC. We believe having this set of SMS-specific guidelines (e.g., engaging a CAB, inter-rater translation methods, technology development and testing), that have been developed from mostly first-hand experience and also build upon extant literature, offer an effective “checklist” of sorts for other researchers planning to integrate SMS into HBC programs in LMICs.

The case study was conducted in a very specific international population (i.e., Xhosa-speaking Black Africans living in Khayelitsha, Cape Town, SA), so we recognize that not all of the situationally-specific barriers faced and decisions made by the *Lifestyle Africa* research team will translate to all other contexts. Nonetheless, this report contributes to a growing body of literature around the potential of mobile technology for improving HBC program implementation, expanding the reach of interventions by providing additional program supports to participants, and using SMS as a method for enhancing participant motivation and self-efficacy for HBC.

## Data Availability

We will make data collected available only upon request from users who can show proof of human subject’s training and only under a data-sharing agreement that provides for: (1) use of the data only for research purposes, (2) exclusion of any identifying or potentially identifying information in shared analyses, publications, reports, etc., (3) appropriate storage and securing of the data to prevent authorized persons from accessing it, (4) a commitment to destroy or return the data after analyses are completed. Inquiries can be sent to institutional administrators at the following email address: ude.hmc@yriuqni-IRC.

## Abbreviations

LMIC: Low- and middle-income country
HBC: Health behavior change
DPP: Diabetes Prevention Program
mHealth: Mobile health
SMS: Short message service/text messaging
MAPS: MHealth Assessment and Planning for Scale Toolkit
WHO: World Health Organization
2G: Second-generation
3G: Third-generation
SA: South Africa/South African
CHW: Community Health Worker
Apps: Mobile applications
CAB: Community advisory board
API: Application programming interface

## References

[1] Diabetes Prevention Program Research Group (2009). 10-year follow-up of diabetes incidence and weight loss in the Diabetes Prevention Program Outcomes Study. Lancet.

[2] Knowler WC, Barrett-Connor E, Fowler SE, Hamman RF, Lachin JM, Walker EA (2002). Reduction in the incidence of type 2 diabetes with lifestyle intervention or metformin. N Engl J Med.

[3] AuYoung M, Moin T, Richardson CR, Damschroder LJ (2019). The diabetes prevention program for underserved populations: a brief review of strategies in the real world. Diabetes Spectr.

[4] Poushter J, Bishop C, Chwe H. Smartphone ownership and internet usage continues to climb in emerging economies. PEW Research Center; 2018. Available from: https://www.pewresearch.org/global/2018/06/19/2-smartphone-ownership-on-the-rise-in-emerging-economies/#:~:text=Smartphone%20ownership%20has%20been%20on,attainment%20in%20every%20country%20surveyed.

[5] Taylor K, Silver L (2019). Smartphone ownership is growing rapidly around the world, but not always equally.

[6] Cole-Lewis H, Kershaw T (2010). Text messaging as a tool for behavior change in disease prevention and management. Epidemiol Rev..

[7] Labrique AB, Wadhwani C, Williams KA, Lamptey P, Hesp C, Luk R (2018). Best practices in scaling digital health in low and middle income countries. Global Health.

[8] Labrique AB, Vasudevan L, Kochi E, Fabricant R, Mehl G (2013). Mhealth innovations as health system strengthening tools: 12 common applications and a visual framework. Glob Heal Sci Pract.

[9] Arigo D, Jake-Schoffman DE, Wolin K, Beckjord E, Hekler EB, Pagoto SL (2019). The history and future of digital health in the field of behavioral medicine. J Behav Med.

[10] WHO (2015). The MAPS toolkit: mHealth assessment and planning for scale.

[11] Catley D, Puoane T, Tsolekile L, Resnicow K, Fleming K, Hurley EA, et al. Adapting the diabetes prevention program for low and middle-income countries: protocol for a cluster randomised trial to evaluate “Lifestyle Africa.” BMJ Open. 2019;9(11):e031400. 10.1136/bmjopen-2019-031400 PMC6858109 31719084

[12] Catley D, Puoane T, Goggin K, Tsolekile LP, Resnicow K, Fleming K (2020). Adapting the diabetes prevention program for low- and middle-income countries: preliminary implementation findings from lifestyle Africa. Transl Behav Med.

[13] Catley D, Puoane T, Tsolekile L, Resnicow K, Fleming KK, Hurley EA (2022). Evaluation of an adapted version of the diabetes prevention program for low- and middle-income countries: a cluster randomized trial to evaluate “Lifestyle Africa” in South Africa. PLoS Med.

[14] Patel S, Shah V, Kansara M (2018). Comparative study of 2G, 3G and 4G. Int J Sci Res Comput Sci Eng Inf Technol.

[15] Campbell JI, Aturinda I, Mwesigwa E, Burns B, Santorino D, Haberer JE (2017). The Technology Acceptance Model for Resource-Limited Settings (TAM-RLS): a novel framework for mobile health interventions targeted to low-literacy end-users in resource-limited settings. AIDS Behav.

[16] Anderson M. Technology device ownership. PEW Research Center; 2018. Available from: https://www.pewresearch.org/internet/2015/10/29/technology-device-ownership-2015/.

[17] Materia FT, Miller EA, Runion MC, Chesnut RP, Irvin JB, Richardson CB (2016). Let’s get technical: enhancing program evaluation through the use and integration of internet and mobile technologies. Eval Program Plann.

[18] Dale LP, Whittaker R, Jiang Y, Stewart R, Rolleston A, Maddison R (2015). Text message and internet support for coronary heart disease self-management: results from the Text4Heart randomized controlled trial. J Med Internet Res.

[19] Mbuagbaw L, Mursleen S, Lytvyn L, Smieja M, Dolovich L, Thabane L (2015). Mobile phone text messaging interventions for HIV and other chronic diseases: an overview of systematic reviews and framework for evidence transfer. BMC Health Serv Res.

[20] Mlambo CK, Vernooij E, Geut R, Vrolings E, Shongwe B, Jiwan S (2019). Experiences from a community advisory board in the Implementation of early access to ART for all in Eswatini: a qualitative study. BMC Med Ethics.

[21] Kreuter MW, Wray RJ (2003). Tailored and targeted health communication: strategies for enhancing information relevance. Am J Health Behav.

[22] Kumar S, Nilsen WJ, Abernethy A, Atienza A, Patrick K, Pavel M (2013). Mobile health technology evaluation: the mHealth evidence workshop. Am J Prev Med.

[23] Noar S, Benac C, Harris M (2007). Does tailoring matter? Meta-analytic review of tailored print health behavior change interventions. Psychol Bull.

[24] Rimer B, Kreuter M (2006). Advancing tailored health communication: a persuasion and message effects perspective. J Commun.

[25] Green J, Thorgood N (2003). Qualitative methods for health research. Sociol Res Online.

[26] Kwasnicka D, Dombrowski S, White M, Sniehotta F (2016). Theoretical explanations for maintenance of beahvior change: a systematic review of behaviour theories. Health Psychol Rev.

[27] Ajzen I (1991). The theory of planned behavior. Organ Behav Hum Decis Process.

[28] Rosenstock IM (1974). The health belief model and preventive health behavior. Health Educ Monogr.

[29] Hall IJ, Johnson-Turbes A (2015). Use of the persuasive health message framework in the development of a community-based mammography promotion campaign. Cancer Causes Control.

[30] Saghafi-Asl M, Aliasgharzadeh S, Asghari-Jafarabadi M. Factors influencing weight management behavior among college students: an application of the Health Belief Model. PLoS One. 2020;15(2):e0228058. 10.1371/journal.pone.0228058. 10.1371/journal.pone.0228058 PMC7006943 32032376

[31] Mobasheri N, Ghahremani L, Abarghooee EF, Hassanzadeh J. Lifestyle intervention for patients with nonalcoholic fatty liver disease: a randomized clinical trial based on the theory of planned behavior. Biomed Res Int. 2022;12(2022):3465980. 10.1155/2022/3465980. 10.1155/2022/3465980 PMC9484896 36132088

[32] Schilling L, Bennett G, Sheana B, Kempe A, Prahl Wretling M, Staton E (2013). Text messaging in healthcare research toolkit.

[33] Mummah SA, Robinson TN, King A, Gardner CD, Sutton S (2016). IDEAS (Integrate, Design, Assess, and Share): a framework and toolkit of strategies for the development of more effective digital interventions to change health behavior. J Med Internet Res.

[34] Schnall R, Rojas M, Bakken S, Brown W, Carballo-Dieguez A, Carry M (2016). A user-centered model for designing consumer mobile health (mHealth) applications (apps). J Biomed Inform.

